# Bibliographical Notice

**Published:** 1840-02-01

**Authors:** 


### BIBLIOGRAPHICAL NOTICE.

*Elements of Pathological Anatomy, illustrated by numerous Engravings.* By Samuel D. Gross, M. D., late Professor of General Anatomy, Physiology, and Pathological Anatomy, in the Medical Department of the Cincinnati College. In two volumes. 8vo. pp. 518, 510. Boston, 1839.

We have no better evidence of the importance attached to the study of pathological anatomy as an aid to diagnosis and treatment, than the publication of these volumes. Much as this department of science has been cultivated in the United States, but a single work has been written expressly upon the subject, that of Dr. Horner. This contains much excellent matter, but is scarcely a complete treatise; nor are the European publications at all adequate to supply this deficiency. Andral, and other writers upon the subject, have published valuable works, replete with facts, but still not complete in themselves, nor competent to answer many questions which the student in pathology is obliged to ask. The best of these works is undoubtedly that of Dr. Carswell; but this is rather an earnest of what the author is capable of doing, than a finished production: it is invaluable for its illustrations, and the admirable descriptions of particular lesions, but not sufficiently extended to admit of the study of the lesions of the organs as connected with the functions of life. Dr. Hope's work is nearly similar to that of Dr. Carswell; it is more extended, but less perfect in its kind.

These works are not all within the reach of the American student: the expense of some of them, the rarity of others, render them nearly useless for purposes of every day inquiries.

We are not disposed to discuss the question of the utility of pathological anatomy. That it may be, and sometimes is abused,—that conclusions are drawn from it to an unwarrantable extent, no one can deny; but that, under proper restrictions, it is a most useful instrument—a most efficient explorer of concealed mischief, giving clearness to our ideas, and a just conception of the disorder caused by diseased action—no reflecting mind can doubt. The reason of the causeless neglect of pathological anatomy by many physicians, is extremely plain. It is a troublesome, often a disgusting pursuit; it is incompatible with the occupations of some physicians, and shocks the indolence, or the extreme sensitiveness of others. But it is neither just, nor consistent with a generous devotion to science, to undervalue, and affect to despise a laborious method of investigation, which is not the ultimate object of medical science, but is an invaluable assistance in our progress, and contributes largely and surely towards this end.

Dr. Gross is already well known as the author and translator of several works of merit and research. Circumstances favoured the cultivation of pathological anatomy, to which he has sedulously devoted himself for some years. The facilities at his command, if somewhat limited, have been much increased by the aid of his professional brethren; and he has well availed himself of these advantages, by a careful examination of the lesions of organs, and collection of the observation of most writers upon pathological anatomy. With the additions to this subject made by American writers, he is not so familiar. It is, however, difficult to incorporate in a work of this kind, the scattered facts and notices which must be gathered from journals extending through a long series of years; and the inductions and critical research of the author are so very creditable to his erudition and love of truth, that we would not notice his trivial omission in an uncharitable spirit.

The leading characters of the work, taken as a whole, are, that it is clearly written, systematic, correct, and more complete than other works upon the subject. Its defects are, that it is occasionally incorrect in its pathology, and does not sufficiently point out those changes which are evidently the secondary lesions of disease, occurring late in its course, and often remaining after the morbid action has ceased.

It is true, that a work on pathological anatomy cannot be required to embrace the whole history of diseased action; but it should include so much of it, as may prevent the student from confounding the lesions of organs with diseased functions, the essential alterations of disease, with those that are accidental and irregular. Dr. Gross has done something towards this, but he has not done enough for the student; the chance of error, in this respect, should be avoided,—for the only objection against pathological anatomy which has the shadow of an argument, is, that when imperfectly studied, it may tend to confound two distinct things—morbid action and morbid effect.

Our readers will perceive, by an analysis of the work, that these criticisms weigh very lightly in the balance against the merits of the work: its accuracy and comprehensive character place it amongst the most valuable additions to medical literature.

A systematic treatment on pathological anatomy is naturally divided into two parts,—one treating of the lesions in an abstract manner, without especial reference to particular organs; and the second containing the examination of the diseased organs themselves. This mode Dr. Gross has followed. After treating of inflammation, he describes its consequences—the effusion of serum, lymphization, suppuration, hæmorrhage, softening, gangrene, ulceration, granulation, cicatrization, induration, hypertrophy, atrophy. Under separate chapters he includes the heterologous transformations, hydatids and serous cysts; all of them he is disposed to attribute to inflammation, properly speaking, or to mere modifications of this action,—that is, to inflammation modified by a peculiar diathesis of the individual.

The heterologous formations, (to use the term which Dr. Gross has adopted, including tubercle, melanosis, scirrhus, encephaloid, and the other products known under the name of cancer,) are, of course, treated of in a distinct chapter: of these, the most frequent is tubercle. The cellular tissue is the one primitively affected,—the mucous, however, nearly as often, Dr. Carswell believes more frequently than any other; but in this respect our views coincide with those of Dr. Gross. The relative frequency of tubercles is compiled from the tables of Drs. Louis and Lombard, which determine the fact that tubercles in adults are much more frequent in the lungs than in any other organ; in children nearly as frequent, but rather less so than in the bronchial glands. The chemical analysis of tubercle, according to Hercht, consists of nearly equal parts of albumen, fibrine, and gelatine. Dr. Gross reconciles this statement with the analysis of Thenard and others, who have detected a large proportion of earthy salts, by the simple fact that this product varies extremely at different periods of its growth. It is, in fact, just as absurd to speak of the analysis of tubercle without reference to its period, as it would be to describe the bone of the fœtus as chemically identical with that of the adult. The tuberculous matter is found in several forms,—the miliary, encysted, infiltrated, and stratiform,—the latter being the variety in which it is deposited upon the surface of membranes. The connexion of tubercle, in its forming stage, with hydatids, is evidently erroneous; but what, then, is the nature of the process by which they are secreted? Dr. Gross ascribes them to inflammatory action: with this opinion our own coincides under some circumstances.

This subject we have studied with extreme care, and lay most stress upon our examinations of tubercles of serous membranes, because in these situations the process may be very accurately traced from the transparency of the tissues, and in the same parts the deposit of this substance is evidently most rapid and acute. In these membranes, particularly in the pleura and peritoneum, the tuberculous matter is deposited in small grains, which are surrounded by a distinct ring of vessels filled with bright arterial blood. The vessels seem to be supplied by others which radiate from, or rather towards the ring. There is no trace of red vessels in the tuberculous granulations themselves, even when examined by the aid of the microscope. The nutrition is then either by a process of inhibition, or by vessels carrying only the transparent fluids. The yellowish tint of the tubercles in jaundice is mentioned by several writers; this is regarded by Dr. Gross as a proof of the organization of tubercles. In the tubercles of the liver the same tinge always exists; a fact, we believe, not before mentioned,—but from the examination of a large number of tuberculous children, we have ascertained this with great certainty.

The process of softening of tubercles, is still almost a mooted point. Dr. Gross explains it by the irritation set up in the surrounding tissues from the presence of the tubercle as a foreign body,—this is transmitted to the tubercle itself, which, possessing an inferior vitality, soon breaks down into a substance closely analogous to pus. A portion of the softened mass undoubtedly consists of nearly pure pus. This explanation is nearly satisfactory. There is, however, a process of rapid softening, which is closely analogous to gangrene, and begins at the centre of the tubercle. Even this process must require a percolation of fluids through the mass, although there is no distinct circulation, nor any proper inflammatory action. The discharge of the softened tuberculous matter, by ulceration, into the adjacent mucous tubes, is well known. The transformation of some tubercles into a dry, concrete, earthy mass, is alluded to by Dr. Gross but slightly.

Melanosis, another of these anomalous products, is described in its three varieties—the tuberoid, lamellated, and dot-like. It is more indolent, and less inclined to soften and discharge itself by ulceration than tubercle.

Scirrhus is defined, by our author, to be "a hard, crisp, opaque substance, of a lightish gray colour, with dull yellowish fibrous intersections, organized, liable to lancinating pain, occurring for the most part after the middle period of life, and passing sooner or later to ulceration." In the tuberoid variety, the morbid substance is deposited in the form of nodules in the interstitial cellular tissue of organs: when diffused through the parenchyma of organs, Dr. Gross considers the term identical with the lardaceous tissue of the French, in which the whole tissue becomes like lard. Scirrhus softens much like tubercle, and ulcerates, forming, as is well known, a troublesome sore. At this period, only, the disease is attended with much pain. Dr. Gross regards scirrhus as caused by inflammatory action. In some cases of diffused scirrhus of the hollow viscera, especially the stomach, this is evident; but in other situations the tuberoid scirrhus is not evidently connected with inflammation,—on the contrary, it is evidently a deposit from the blood, arising from a peculiar alteration of this fluid.

The term encephaloid is used with some extension of its usual signification. He classes under this title the various diseases known sometimes under the generic appellation, soft cancer. These are medullary sarcoma, fungus hæmatodes, soft cancer, medullary fungus, and spongoid inflammation. The term encephaloid is usually restricted to one variety of these diseases; and, in this respect, we think the ordinary classification better than the nomenclature of Dr. Gross. It is very evident that the term encephaloid is applicable only to that variety of the disease which is closely analogous to brain in its structure, and even in its chemical properties: while the term soft cancer includes every variety, however unlike they may be in composition. The dependence of encephaloid upon a general morbid diathesis, and not on local inflammation, is admitted by Dr. Gross.

The study of special pathological anatomy is appropriately introduced by the morbid alterations of the blood. The author has given all that is positively known upon the subject, but we are constantly compelled to regret the barrenness of his materials. Strange to say, evident as are the alterations of the blood, we are able to say little more respecting them than that the quantity of fibrine and of red globules is increased in inflammatory diseases, diminished in anemia, and all morbid conditions in the nutrition is affected,—while in many cases of poisoning, and in some malignant fevers, the crasis is broken down, and the colour of the venous and arterial blood is much darker than usual. The fatty serum, and dark wheylike serum, are conditions occurring in inflammation, particularly those connected with a morbid nutrition.

The tissues of the body are subject to the diseases already alluded to, which give rise to various alterations, according to the structure in which they are seated; hence Dr. Gross has very properly taken up the consideration of these alterations before proceeding to the lesions of particular organs. This portion of the work is remarkably complete, especially that relative to the diseases of the skin, which includes a complete classification of these disorders.

After the pathological anatomy of the tissues, the author passes to the lesions of the brain and its envelopes, including, of course, meningitis. His description of this disease is somewhat less satisfactory than other portions of his work, especially those relative to the pathology of the tissues. Of the variety of disease known under the name of tuberculous meningitis, he has given no complete description: this is the acute hydrocephalus of young children, and arises from an inflammatory disease of the membranes of the brain, connected with a deposit of tuberculous granulations. There are usually more or less evident alterations of the brain itself, and effusion of serum into the ventricles, or beneath the arachnoid. These lesions modify more or less the symptoms of the disorder; but in the essential part it is still the same,—that is, a tuberculous disease. In nearly every case the nature of the disorder is rendered more evident by the appearance of tubercles in other organs than the brain.

Under the morbid anatomy of the lungs phthisis is included, although this disease is so extended in its action that many organs, besides the lungs, are more or less involved. These are well described by Dr. Gross, who has availed himself of the numerous additions made of late years to our knowledge of this subject.

The inflammation of the pericardium is accurately described: the symptoms of the disease are also given, but with less clearness than could have been desired. The endocardium, which is so frequently the seat of inflammation, and gives rise to many chronic diseases of the valves of this organ, is rather overlooked; its alterations, and their terminations, are not described as accurately as the importance of the disease requires. The additions to our knowledge of heart diseases depend mainly upon the more perfect acquaintance with the alterations of the endocardium.

The gastric and abdominal viscera are so important in their connexions with the system in general, and so subject to various marked changes, that they necessarily occupy a large place in a work on pathological anatomy. The descriptions are good, and as complete as in other portions of the work. The defect lies rather in the pathology, than in the morbid anatomy. The subject of the lesions of the follicles of the small intestines naturally belongs to this portion of the work; and its connexion with typhoid fever is necessarily alluded to. The author has done justice to the labours of the French pathologists in this respect; but he has not stated that the question of the distinction between the typhus of the English writers, and the typhoid fever of the French, has been settled by the investigations of the two diseases at the Philadelphia Hospital. Both of these diseases may exist at the same time,—but their progress and symptoms are quite as different as those of measles and scarlet fever; and in the one there is disease of the follicles of the small intestines, while in the others these bodies are perfectly normal.

Typhoid fever is sometimes latent: of this an interesting case occurred at Cincinnati, which is mentioned by Dr. Gross. Death occurred from perforation, in consequence of the ulcer seated near the ileum, and in all probability dependent upon a disease of a gland of Peyer. This case is mentioned as ulceration of the ileum.

The description of the other lesions of the stomach and small intestines is highly interesting, from its variety and accuracy, and may be consulted with great advantage by the practitioner.

The colon is a frequent seat of inflammation and ulceration, like the small intestine, and certainly recovers, as well as any other portion of the alimentary canal, from these alterations. Dr. Gross believes that "it is a singular fact, and one for which he cannot offer any satisfactory explanation, that ulcers of the large bowel are much less liable to cicatrize than those of the small. Never, indeed, in a single instance, have I seen complete reparation in this situation, though it has occurred to me on several occasions to notice partial efforts of this sort." This error of Dr. G. has evidently arisen from his confounding tuberculous ulceration, in which the follicles contain tuberculous matter, which softens and is discharged, with ordinary acute inflammatory ulcers. In the former variety, it is true, complete reparation is very rare,—in the latter it is exceedingly frequent. This error was very natural for one who has chiefly examined the bodies of patients in private practice, in which it is extremely difficult to investigate the alterations of the mucous coat of the bowels in a satisfactory manner. But we have very often seen, in hospital practice, every stage of cicatrization of the large bowel, from the first subsidence of the edges of the ulcer, to the smooth, shining cicatrix. A number of these patients died of various chronic diseases, many months after their recovery from some dysentery. In process of time the cicatrix becomes gradually less and less visible, and the newly-formed mucous coat scarcely differs from the inguinal tissue. In all cases these cicatrices are less puckered than in other portions of the bowels, and therefore more difficult to recognise.

An excellent account of intestinal worms will be found in the work; and of the descriptions of the lesions of the peritoneum we can also speak in terms of unqualified praise.

The lesions of the liver are, it is well known, extremely common. But they may be classed under a few heads. Inflammation and its terminations, including suppuration, hypertrophy of the acini, with change of colour, known under the name of cyrrhosis and atrophy. These all result from acute or chronic inflammation, or at least chronic irritation. The hypertrophy of the whole organ depends sometimes upon chronic irritation, at others upon a peculiar diathesis. The tuberculous and cancerous formations in the liver seem to be totally independent of any local cause. All these lesions are described briefly, but with clearness and accuracy.

The few lesions of the spleen and pancreas occupy but small space, the induration of the spleen in chronic intermittent, and its softening in intermittent, as well as other fevers, are its chief disorders. These alterations do not seem always to depend upon inflammation, to which they are referred by Dr. Gross. In some cases, at least, the softening depends upon the altered condition of the blood.

The kidneys are regarded as directly connected with certain pathological states of the system, since the publication of Dr. Bright's researches upon the granular disease of the kidneys,—a frequent cause of dropsy. This condition is, with reason, regarded by our author as closely analogous to cyrrhosis of the liver. The acute inflammation of the kidneys, and its calculous deposits are well known.

The male organs of generation are the seat of many alterations, which receive their due share of attention, with one exception, the chronic inflammation of the vesiculæ seminales; a condition which not unfrequently gives rise to diurnal or involuntary discharges of semen, and often indirectly undermines the health. This condition is described by Professor Lallemand, who has probably attached an exaggerated importance to it.

The female organs of generation are treated of in the concluding chapter of the book. Their alterations are frequent, but for the most part extremely simple; they are noticed by Dr. Gross, without entering into the minute details of the slighter lesions.

The work is illustrated, by nearly one hundred excellent wood-cuts, and a coloured engraving of the inflamed mucous membrane of the alimentary canal. The illustrations are therefore as abundant as is compatible with a price which would put the book in the hands of the profession in general. It deserves an extended circulation, not only for its own merits, but because no other work, now in the hands of the profession, can supply its place. In proportion as medicine is regarded as a comprehensive science, it will be necessary to observe the alterations of structure, as well as the deviations of the functions from a healthy standard. No evil can result from a thorough knowledge of pathological anatomy, although a superficial acquaintance with the subject may lead to error. We have spoken in high terms of the work of Dr. Gross, because it possesses real and durable excellencies; these form the leading characteristics of it. Its omissions and errors are so limited in number that we have had little difficulty in pointing out all those which are of much importance. To a certain extent they are inevitable in so comprehensive a work, but some of them would doubtless have been corrected, if the author had been able to publish the work under his immediate revision. As it is, they do not impair the character of the work, or prevent it from ranking among the standard productions of medical literature.

---

*The American Medical Almanac, for* 1840, *designed for the daily use of practising physicians, surgeons, students, and apothecaries. Being also a Pocket Memorandum and Account Book, and General Medical Directory of the United States and the British Provinces.* By J. V. Smith, M. D., Editor of the Boston Med. and Surg. Journal. Boston, 1840: 18 mo., pp. 152.

By some accident, this work has just reached us. It contains an immense amount of useful information to the physician. The undertaking is still novel, this being only the second year, and cannot, therefore, be regarded as a fair test of the future correctness of the work. In a subsequent edition we have no doubt that some errors and omissions which have crept into the present one, will be avoided. We allude particularly to the account of the institutions at Philadelphia. At the same time, we think it but justice to the author to state that a portion of them would have been avoided, if the information which he had expected, had been forthcoming at an early period. We can promise our aid in preventing a similar deficiency in future editions, which we have no doubt will be regularly called for.

